# Brunsting-Perry Pemphigoid as Differential Diagnosis of Nonmelanoma Skin Cancer

**DOI:** 10.7759/cureus.5400

**Published:** 2019-08-16

**Authors:** Gerhard Eichhoff

**Affiliations:** 1 Dermatology Service, Wellington Regional Hospital, Wellington, NZL

**Keywords:** brunsting-perry pemphigoid, cicatricial pemphigoid, epidermolysis bullosa acquisita, bullous pemphigoid, squamous cell carcinoma

## Abstract

Brunsting-Perry pemphigoid is a rare autoimmune blistering skin disease. Similar to nonmelanoma skin cancers, Brunsting-Perry pemphigoid has a predilection for the head and neck. Herein, a case of solitary Brunsting-Perry pemphigoid treated as cutaneous squamous cell carcinoma (SCC) with subsequent excision is reported.

## Introduction

Brunsting-Perry pemphigoid, an autoimmune blistering disease with only about 60 reported cases, is considered a subtype of either cicatricial pemphigoid, bullous pemphigoid or epidermolysis bullosa acquisita [1]. Furthermore, Brunsting-Perry pemphigoid is a heterogeneous disorder manifesting in multiple or solitary lesions with blisters, erosions, crusting, or scarring and affects predominantly the head and neck. As direct immunofluorescence regularly remains negative and peripheral antibodies are rarely detected, the diagnosis of Brunsting-Perry pemphigoid is often made upon clinical and histopathological findings [1-2]. The treatment of Brunsting-Perry pemphigoid consists mainly of topical, intralesional and/or systemic steroids, and steroid-sparing medication [1].

Skin cancer is often managed in primary care and specialties without the involvement of dermatologists [3]. It is common practice to excise lesions suspicious for skin cancer without prior histological confirmation. Several mimickers of nonmelanoma skin cancer, mostly benign tumors, have been described in the literature [4].

## Case presentation

A 76-years old female Caucasian patient without a history of skin cancer was referred from primary care to a secondary hospital with an 8-months history of a tender, sometimes weeping scalp lesion. The topical treatment with fusidic acid cream 2% and miconazole nitrate 2% + hydrocortisone 1% cream improved the lesion partially but it never subsided.

**Figure 1:**
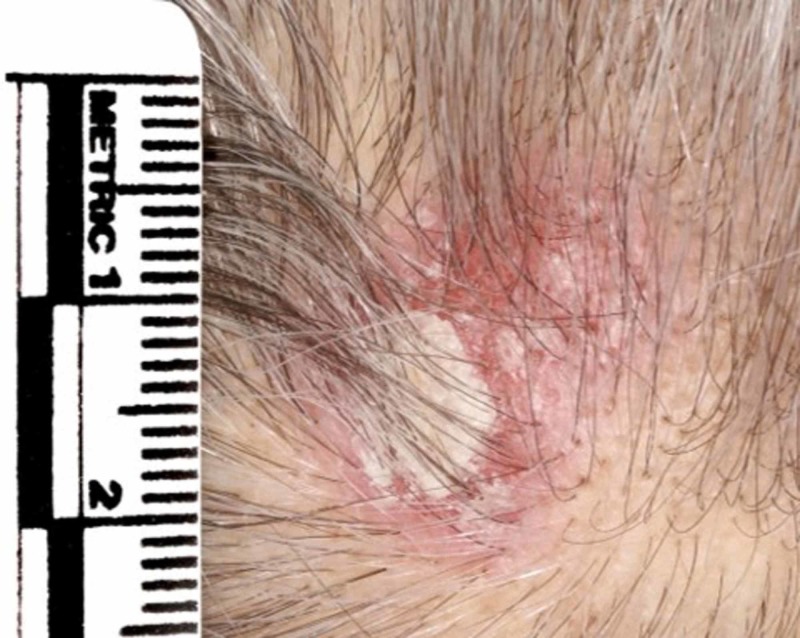
Indurated erythematous plaque with crusting on the vertex of the scalp.

The patient presented to the hospital with an erythematous, crusting and lesion measuring 2 x 2 cm on the vertex of the scalp (Figure 1).

SCC was suspected, and the lesion was removed completely with subsequent split skin grafting.

**Figure 2:**
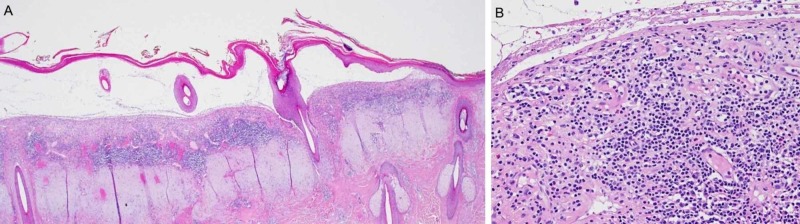
(A) Subepidermal blister formation with band-like dermal inflammatory cell infiltrate (HE stain x 20). (B) Close-up of denuded dermis with the scattering of eosinophils (HE stain x 200).

Histopathology revealed a subepidermal blister with a chronic band-like inflammatory cell infiltrate with scattered eosinophils in the dermis without any sign of malignancy (Figure 2).

Three months later, the patient was referred to the Dermatology department, where a well-healed scar without any pathological findings was noticed. At this stage, direct immunofluorescence of the lesion could not be requested, as it was excised completely and formalin fixed, auto-antibodies to BP180 and BP230 were not be detected in the patient’s blood. The retrospective diagnosis of Brunsting-Perry pemphigoid was made after taking into consideration the patient’s history, the clinical manifestation, and the pathological findings.

## Discussion

Brunsting-Perry pemphigoid is an unusual differential diagnosis of nonmelanoma skin cancer. Monihan et al. reported three cases of Brunsting-Perry pemphigoid simulating superficial basal cell carcinomas [5]. Especially, in the setting of a solitary, scaly lesion, Brunsting-Perry pemphigoid can be mistaken for nonmelanoma skin cancer or pre-cancer, which led to the presumptive diagnosis of SCC in the case presented here [2].

## Conclusions

The reported findings highlight the importance of histopathological examination of skin lesions with an unusual history or presentation prior to excision. Additionally, they emphasize that surgeons involved in skin cancer management must have appropriate knowledge about the differential diagnoses of nonmelanoma skin cancers aiming to avoid unnecessary excision.
